# Supplementary material for: Diffusion-based generative AI for exploring transition states from 2D molecular graphs
Source: Nat Commun. 2024 Jan 6;15:341. doi: 10.1038/s41467-023-44629-6 (PMC10771475; doi:10.1038/s41467-023-44629-6)
Supplement: Supplementary file 1 — Supplementary Information [file 41467_2023_44629_MOESM1_ESM.pdf]

Supplementary Information for:

# Diffusion-based Generative AI for Exploring Transition States from 2D Molecular Graphs

Seonghwan Kim,[1†] Jeheon Woo,[1†] and Woo Youn Kim[1,2*]

[1]*Department of Chemistry, KAIST, 291 Daehak-ro, Yuseong-gu, 34141, Daejeon, Republic of Korea.*

[2]*AI Institute, KAIST, 291 Daehak-ro, Yuseong-gu, 34141, Daejeon, Republic of Korea.*

[*]Corresponding author. E-mail: wooyoun@kaist.ac.kr

Contributing authors: dmdtka00@kaist.ac.kr; woojh@kaist.ac.kr

[†]*These authors contributed equally to this work.*

# 1 Supplementary Methods

## Sampling algorithm

The diffusion process is a fundamental element in our proposed method, TSDiff, as it allows for the modeling of data as a sequence of noisy observations. In this section, we provide a brief description of the diffusion process, its reverse process, and the sampling algorithms. Although these discussions have been covered in other papers [1, 2], we include them here for completeness in the description of TSDiff.

Our design choice of the diffusion process is based on the denoising diffusion probabilistic modeling (DDPM) [1], and its forward transition probability and transition kernel are given by,

$$\begin{aligned} q(\mathcal{C}_t|\mathcal{C}_{t-1}) &= \mathcal{N}(\mathcal{C}_t; \sqrt{1-\beta_t}\mathcal{C}_{t-1}, \beta_t \mathbf{I}), \\ q(\mathcal{C}_t|\mathcal{C}_0) &= \mathcal{N}(\mathcal{C}_t; \sqrt{\bar{\alpha}_t}\mathcal{C}_0, (1-\bar{\alpha}_t)\mathbf{I}). \end{aligned} \tag{1}$$

The posterior of this forward diffusion process can be expressed as follows using the Markov chain assumption and Bayes rule:

$$q(\mathcal{C}_{t-1}|\mathcal{C}_t, \mathcal{C}_0) = \frac{q(\mathcal{C}_t|\mathcal{C}_{t-1})q(\mathcal{C}_{t-1}|\mathcal{C}_0)}{q(\mathcal{C}_t|\mathcal{C}_0)}. \tag{2}$$

Since all distributions in right hand side of Supplementary Equation (2) are Gaussian, the posterior distribution is also Gaussian, given by $\mathcal{N}(\mathcal{C}_{t-1}; \mu_t(\mathcal{C}_t, \mathcal{C}_0), \tilde{\beta}_t \mathbf{I})$ with the following parameters:

$$\mu_t(\mathcal{C}_t, \mathcal{C}_0) = \frac{\sqrt{\bar{\alpha}_{t-1}}\beta_t}{1-\bar{\alpha}_t}\mathcal{C}_0 + \frac{\sqrt{\alpha_t}(1-\bar{\alpha}_{t-1})}{1-\bar{\alpha}_t}\mathcal{C}_t \quad \text{and} \quad \tilde{\beta}_t = \frac{1-\bar{\alpha}_{t-1}}{1-\bar{\alpha}_t}\beta_t. \tag{3}$$

The distribution is for $\mathcal{C}_{t-1}$ given $\mathcal{C}_t$, but it is intractable in the sense that it requires an intractable $\mathcal{C}_0$ during the inference phase. Here, we approximate this posterior by modeling the parametric distribution $p_\theta$ as follows:

$$p_\theta(\mathcal{C}_{t-1}|\mathcal{C}_t, \mathcal{G}_{\text{rxn}}) = \mathcal{N}(\mathcal{C}_{t-1}; \mu_\theta(\mathcal{C}_t, t, \mathcal{G}_{\text{rxn}}), \sigma_t^2 I). \tag{4}$$

We used $\sigma_t$ as the same as $\beta_t$ following Ho et al [1]. By re-parameterizing $\mathcal{C}_0$ in the $\mu_t$ formula according to Supplementary Equation (1), we can naturally design $\mu_\theta$ accordingly:

$$\begin{aligned} \mu_t(\mathcal{C}_t, \varepsilon) &= \frac{1}{\sqrt{\alpha_t}}\left(\mathcal{C}_t - \frac{\beta_t}{\sqrt{1-\bar{\alpha}_t}}\varepsilon\right), \\ \mu_\theta(\mathcal{C}_t, t, \mathcal{G}_{\text{rxn}}) &= \frac{1}{\sqrt{\alpha_t}}\left(\mathcal{C}_t - \frac{\beta_t}{\sqrt{1-\bar{\alpha}_t}}\varepsilon_\theta(\mathcal{C}_t, t, \mathcal{G}_{\text{rxn}})\right), \end{aligned} \tag{5}$$

where $\varepsilon = -\frac{\sqrt{\bar{\alpha}_t}\mathcal{C}_0 - \mathcal{C}_t}{\sqrt{1-\bar{\alpha}_t}} \sim \mathcal{N}(\mathbf{0}, \mathbf{I})$. The training loss of the KL divergence between the posterior of $q$ and $p_\theta$ is proportion to $\|\mu_t - \mu_\theta\|^2$, which is translated to $\|\varepsilon - \varepsilon_\theta\|^2$. Therefore, the sampling process based on these is depicted in Supplementary Algorithm 1.

**Supplementary Algorithm 1** DDPM sampling

Input: the reaction graph $\mathcal{G}_{\text{rxn}}$, the trained neural network $\varepsilon_\theta$, the diffusion coefficient $\{\beta_t\}_{t=1}^T$.

```
Draw C_T ~ p(C_T) = N(0, I)
for t = T, ..., 1 do
    μ_θ(C_t, G_rxn, t) ← 1/√α_t ( C_t − β_t/√(1−ᾱ_t) ε_θ(C_t, t, G_rxn) )
    draw z_t ~ N(0, I)
    C_{t−1} ← μ_θ(C_t, G_rxn, t) + σ_t z_t
end for
return C_0
```

On the one hand, since $\varepsilon = -\sqrt{1-\bar{\alpha}_t}\nabla_{\mathcal{C}_t} \log q(\mathcal{C}_t|\mathcal{C}_0)$, training objective can be interpreted as matching the score function of $q(\mathcal{C}_t|\mathcal{C}_0)$. The forward process of TSDiff can be interpreted as an it$\hat{o}$ process of a

variance preserving stochastic differential equation (SDE) that converges to a unit Gaussian [3]. By simply applying a scaling factor $1/\sqrt{\bar{\alpha}_t}$ to $\mathcal{C}_t$, the scaled forward process could be a variance exploding SDE. The $\mathcal{X}_t$ is the scaled random variable from $\mathcal{C}_t$, such that $\mathcal{X}_t = \mathcal{C}_t/\sqrt{\bar{\alpha}_t}$ and $\mathcal{X}_0 = \mathcal{C}_0$. In this case, the transition probability that describes the forward process is defined as follows:

$$\begin{aligned} q(\mathcal{X}_t|\mathcal{X}_{t-1}) &= \mathcal{N}\left(\frac{\mathcal{C}_t}{\sqrt{\bar{\alpha}_t}}; \frac{\sqrt{1-\beta_t}\mathcal{C}_{t-1}}{\sqrt{\bar{\alpha}_t}}, \frac{\beta_t}{\bar{\alpha}_t}I\right) = \mathcal{N}(\mathcal{X}_t; \mathcal{X}_{t-1}, \hat{\sigma}_t^2 I), \\ q(\mathcal{X}_t|\mathcal{X}_0) &= \mathcal{N}(\mathcal{X}_0, \bar{\sigma}_t^2), \end{aligned} \tag{6}$$

where $\hat{\sigma}_t^2 = \frac{\beta_t}{\bar{\alpha}_t}$ and $\bar{\sigma}_t^2 = \sum_{i=1}^t \hat{\sigma}_i^2 = \frac{1-\bar{\alpha}_t}{\bar{\alpha}_t}$. From Supplementary Equation (6), we can re-parameterize it as follows:

$$\begin{aligned} \mathcal{X}_t &= \mathcal{X}_{t-1} + \hat{\sigma}_t^2 \mathbf{z}_t, \\ \mathcal{X}_t &= \mathcal{X}_0 + \bar{\sigma}_t^2 \bar{\mathbf{z}}_t, \end{aligned} \tag{7}$$

where $\bar{\mathbf{z}}_t, \mathbf{z}_t \sim \mathcal{N}(\mathbf{0}, \mathbf{I})$. The $\mathcal{X}_t$ is sampled as the formalism of denoising score matching (DSM) which is a variance exploding case [4–6]. These findings are well aligned, in that $\bar{\alpha}_t$ converges to zero for large $t$. Furthermore, Supplementary Equation (8) ensures that the score function is still invariant under a scaling factor $1/\bar{\sigma}_t$, indicating a score matching model can be shared to simulate the reverse process of both the variance exploding case and the variance preserving case.

$$\begin{aligned} \nabla_{\mathcal{X}_t} \log q(\mathcal{X}_t|\mathcal{X}_0) &= \frac{\mathcal{X}_0 - \mathcal{X}_t}{\bar{\sigma}_t^2} \\ &= \frac{\mathcal{C}_0 - \mathcal{C}_t/\sqrt{\bar{\alpha}_t}}{\bar{\sigma}_t^2} \\ &= \frac{1}{\bar{\sigma}_t} \frac{\sqrt{\bar{\alpha}_t}\mathcal{C}_0 - \mathcal{C}_t}{\sqrt{1-\bar{\alpha}_t}} \\ &= \frac{1}{\bar{\sigma}_t} \nabla_{\mathcal{C}_t} \log q(\mathcal{C}_t|\mathcal{C}_0) \end{aligned} \tag{8}$$

Since the scaled forward process is similar to that of DSM, Langevin dynamics sampling, often used in DSM, is also a natural choice for sampling [4–6]. The Langevin dynamics sampling process requires the score function of the distribution and it can be prepared by scaling the input of the trained score function approximator. The annealed Langevin dynamics sampling process is described in Supplementary Algorithm 2.

**Supplementary Algorithm 2** Annealed Langevin dynamics sampling

Input: the reaction graph $\mathcal{G}_{\text{rxn}}$, the trained neural network $\varepsilon_\theta$, the step size coefficient $c$, the scaling factor $\{\bar{\alpha}_t\}_{t=1}^T$, and the noise level $\{\bar{\sigma}_t\}_{t=1}^T$.

1: Draw $\mathcal{X}_T \sim p(\mathcal{X}_T) \sim \mathcal{N}(\mathbf{0}, \bar{\sigma}_T^2 \mathbf{I})$
2: **for** $t = T, ..., 1$ **do**
3: $\gamma_t \leftarrow c \cdot \bar{\sigma}_t^2$
4: $\mathbf{s}_\theta(\mathcal{X}_t, \mathcal{G}_{\text{rxn}}, t) \leftarrow -\frac{1}{\bar{\sigma}_t\sqrt{1-\bar{\alpha}_t}} \varepsilon_\theta(\sqrt{\bar{\alpha}_t}\mathcal{X}_t, \mathcal{G}_{\text{rxn}}, t)$
5: Draw $\mathbf{z}_t \sim \mathcal{N}(\mathbf{0}, \mathbf{I})$
6: $\mathcal{X}_{t-1} \leftarrow \mathcal{X}_t + \gamma_t \mathbf{s}_\theta(\mathcal{X}_t, \mathcal{G}_{\text{rxn}}, t) + \sqrt{2\gamma_t}\mathbf{z}_t$
7: **end for**
8: **return** $\mathcal{X}_0$

In theory, DSM and DDPM have been proven to be similar methods for solving SDEs, and the scaling trick shown here confirms that they can be considered equivalent with a simple scaling [3]. In other words, we can say that the two sampling methods simulate a diffusion process that produces the same trajectory except for the scaling. The sampling results of both algorithms demonstrated similar accuracy, but there was a slight improvement in the results obtained with Langevin dynamics. As a result, the evaluations of TSDiff including subsequent validation of quantum calculation were conducted using samples obtained by Langevin dynamics.

## 2 Supplementary Discussion

### Application to Birkholz and Schlegel's benchmark

We demonstrate a practical application of TSDiff to identifying the most energetically favorable TS, in conjunction with a clustering algorithm. To evaluate our approach, we used a benchmark set created by Birkholz and Schlegel [7], which consists of chemical reactions commonly used to assess conventional TS discovery methods. This benchmark set contains a total of 20 different chemical reactions. For our experiments with TSDiff, we focused on 13 reactions characterized by a neutral charge and composed of the elements C, H, O, and N, taking into account the training domain of TSDiff. For the consistency of our study, we obtained reference TS geometries for the reactions by TS optimization based on density functional theory at the level of $\omega$B97X-D3/def2-TZVP, using the given geometries from the benchmark as a starting point.

The geometries generated by TSDiff exhibit a clustering tendency based on their conformation. This character suggests the possibility of efficient TS conformational search without the need to perform quantum chemical calculations on the entire set of generated samples. Consequently, this allows an effective selection of TS candidates and reduces the number of quantum calculations required. For each reaction, our experiments were performed along the following procedure: First, we generated one hundred samples using TSDiff. Then, we used Ward's method [8] to identify several cluster sets by grouping samples with similar conformations. Here, we utilized interatomic distances as a clustering feature, and cluster distances were calculated using the Euclidean norm, with an atom index alignment to prevent mismeasurement by index permutation among indistinguishable nodes within the molecular graph. For subsequent quantum chemical calculations, we randomly selected up to two samples from each cluster formed by more than three sample components. We then performed TS optimization based on the Berny algorithm [9] for the selected samples, where the Hessian calculation was performed only once in the first step of the TS optimization. Supplementary Table 1 shows, for each reaction, the number of clusters obtained by Ward's method and the results of the subsequent quantum chemical calculations. For most of the reactions, the TS conformation corresponding to the reference geometry was found, demonstrating the transferability of TSDiff.

Supplementary Table 1: **Transition state (TS) optimization results based on TS conformers generated by TSDiff.** For each reaction in Birkholz and Schlegel's benchmark [7], the number of clusters and optimized TSs obtained, whether the reference TS conformation is included in the optimized TSs, and the number of force calls in the TS optimization computations averaged over clusters are shown.

| reaction | index | # of cluster | # of TS | ref included? | # of forces (avg.) |
|---|---|---|---|---|---|
| $C_2N_2O$ | 1 | 1 | 1 | Y | 15 |
| $C_5HT$ | 2 | 3 | 3 | Y | 27.00 |
| HCN | 3 | 1 | 1 | Y | 3 |
| Cope | 4 | 2 | 2 | Y | 5.00 |
| CPHT | 5 | 1 | 1 | Y | 6 |
| Cyc-But | 6 | 1 | 1 | N | 33 |
| DACP2 | 7 | 2 | 2 | Y | 24.00 |
| DACP+eth | 8 | 1 | 1 | Y | 7 |
| Ene | 10 | 1 | 1 | Y | 8 |
| $H_2$+CO | 12 | 1 | 1 | Y | 11 |
| Hydro | 14 | 4 | 4 | N | 42.25 |
| MeOH | 15 | 1 | 1 | Y | 4 |
| OxyCope | 17 | 2 | 2 | Y | 4.50 |
| average | | | | | 14.60 |

For some reactions involving reaction conformers, TSDiff demonstrated the ability to explore multiple TS conformations. As an example, Supplementary Figure 1 shows the clustering results for the OxyCope and DACP2 reactions. Two distinct clusters were identified for both reactions, and the t-distributed stochastic neighbor embedding (t-SNE) plots effectively depict the clustering characteristics. The dendrograms display the clustering results obtained by Ward's method, an algorithm for hierarchical cluster analysis. Given its hierarchical nature, the number of clusters can vary depending on the threshold chosen. In these two

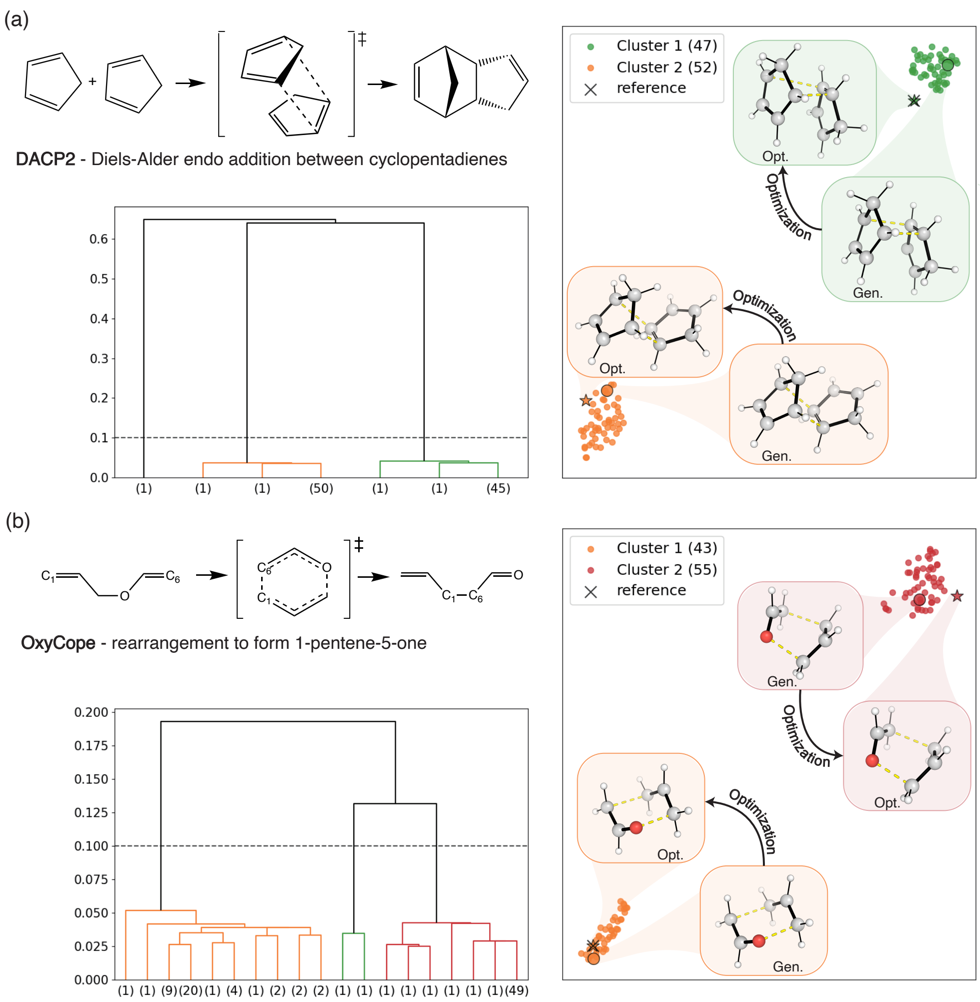


Supplementary Figure 1: **Visualization of clustering: t-distributed stochastic neighbor embedding (t-SNE) and dendrogram analysis of (a) DACP2 and (b) OxyCope.** The dendrograms show the hierarchical clustering results obtained by Ward's method [8]. The dots indicate the geometries generated by TSDiff for the given reaction above. An example of the generated TS conformation of each cluster is also plotted. All molecular geometries were plotted using PyMOL [10].

reactions, a threshold value of 0.1 was applied.

The generated geometries of randomly selected samples from each cluster and their corresponding optimized geometries are shown in the insets of the t-SNE plots. In the DACP2 reaction, the product molecule is formed in a Diels-Alder reaction between two cyclopendadienes. TSDiff has identified two distinct TS conformations, each associated with a different reaction pathway leading to endo and exo products, which are stereoisomers with identical molecular connectivity. In the OxyCope reaction, two different conformations are observed, characterized as boat and chair forms. For example, in the t-SNE plot of Supplementary Figure 1b, the reference TS, marked with a cross, corresponds to the boat-like conformation and is overlapped in agreement with its optimized result, marked with an asterisk. Another conformation discovered by TSDiff is the chair form. It is also noteworthy that the chair conformation has a lower energy than the boat conformation, highlighting the importance of conformational search in TS exploration as emphasized in the main text.

To illustrate the efficiency of TS search process using TSDiff, we present the number of force calls in the TS optimization based on the Berny algorithm in Supplementary Table 1. Furthermore, in Supplementary Table 2, we compare the number of force and Hessian calls with those of the EIP [11] and i-EIP [12] methods,

Supplementary Table 2: **Comparison of TSDiff, EIP [11], and i-EIP [12] methods.** For the reactions in Birkholz and Schlegel's benchmark [7], the number of force and Hessian calls of transition state (TS) optimization computations are compared. The values of EIP and i-EIP are borrowed from [12].

| | | # of forces | | | # of Hessians | | |
|---|---|---|---|---|---|---|---|
| reaction | index | TSDiff | EIP | i-EIP | TSDiff | EIP | i-EIP |
| $C_2N_2O$ | 1 | 15 | 55 | 31 | 1 | 1 | 2 |
| $C_5HT$ | 2 | 5 | 50 | 29 | 1 | 1 | 2 |
| HCN | 3 | 3 | 25 | 26 | 1 | 1 | 1 |
| Cope | 4 | 5 | 97 | 102 | 1 | 1 | 2 |
| CPHT | 5 | 6 | 51 | 26 | 1 | 1 | 2 |
| DACP2 | 7 | 39 | 96 | 68 | 1 | 1 | 2 |
| DACP+eth | 8 | 7 | 56 | 48 | 1 | 1 | 2 |
| Ene | 10 | 8 | 160 | 84 | 1 | 1 | 2 |
| $H_2$+CO | 12 | 11 | 61 | 38 | 1 | 1 | 1 |
| OxyCope | 17 | 4 | 81 | 103 | 1 | 1 | 2 |
| average | | 10.3 | 73.2 | 55.5 | 1 | 1 | 1.8 |

which are recently introduced TS finding methods based on a double-ended approach. Both of these methods utilize atomic forces and Hessians obtained by quantum calculations with reactant and product geometries as input to locate the TS geometry. It's worth noting that the values for EIP and i-EIP in the table are sourced from their original paper [12], which employed density functional theory calculations at the B3LYP-D3/6-31G* level. The results for the MeOH reaction have been omitted as they are not available in the original paper. For a fair comparison for the same reaction conformer, the TS optimization results corresponding to the reactions for which TSDiff found the reference TS conformation are included in the comparison. Finally, the results are compared across a total of 10 reactions, as shown in Supplementary Table 2.

TSDiff consistently produced the smallest number of force calls for all reactions listed in Supplementary Table 2. While EIP and i-EIP require reactant and product geometries as input and are designed to locate a specific TS conformation, TSDiff generates multiple TS conformations. Hence, it can be challenging to make an equivalent efficiency comparison between them. However, the remarkably low number of force calls required by TSDiff clearly demonstrates its efficiency in TS optimization. Consequently, these results confirm that TSDiff can simplify demanding tasks in input preparation, such as establishing molecular conformations and orientations of reactant and product, while significantly improving the efficiency of the subsequent optimization process through its precise geometry generation.

# 3 Supplementary Notes

## Hyperparameters

We here append all hyperparameters related to TSDiff including training hyperparameters (see Supplementary Table 3).

Supplementary Table 3: **Hyperparameters of TSDiff.**

| parameter | value |
|---|---|
| $\beta_1$ | 1e-7 |
| $\beta_T$ | 2e-3 |
| $\beta$ scheduler | sigmoid |
| $T$ | 5,000 |
| hidden dimension | 256 |
| layers | 7 |
| activation | swish |
| $\tau$ | 10 Å |
| train iters | 400,000 |
| batch size | 200 |
| learning rate | 1e-3 |
| optimizer | Adam |
| sampling method | Langevin |
| Langevin step coefficient | 1e-3 |

# Supplementary References


[1] J. Ho, A. Jain, and P. Abbeel, "Denoising diffusion probabilistic models," (2020).

[2] M. Xu, L. Yu, Y. Song, C. Shi, S. Ermon, and J. Tang, "Geodiff: a geometric diffusion model for molecular conformation generation," (2022).

[3] Y. Song, J. Sohl-Dickstein, D. P. Kingma, A. Kumar, S. Ermon, and B. Poole, "Score-based generative modeling through stochastic differential equations," (2020).

[4] Y. Song and S. Ermon, "Generative modeling by estimating gradients of the data distribution," (2019).

[5] C. Shi, S. Luo, M. Xu, and J. Tang, "Learning gradient fields for molecular conformation generation," (2021).

[6] C. Meng, L. Yu, Y. Song, J. Song, and S. Ermon, "Autoregressive score matching," (2020).

[7] A. B. Birkholz and H. B. Schlegel, "Using bonding to guide transition state optimization," Journal of Computational Chemistry **36**, 1157–1166 (2015).

[8] J. H. Ward, "Hierarchical grouping to optimize an objective function," Journal of the American Statistical Association **58**, 236–244 (1963).

[9] H. B. Schlegel, "Optimization of equilibrium geometries and transition structures," Journal of Computational Chemistry **3**, 214–218 (1982).

[10] Schrödinger, LLC, "The PyMOL molecular graphics system, version 2.0," (2017).

[11] Y. Liu, H. Qi, and M. Lei, "Elastic image pair method for finding transition states on potential energy surfaces using only first derivatives," Journal of Chemical Theory and Computation **18**, 5108–5115 (2022).

[12] Y. Liu, H. Qi, and M. Lei, "Improved elastic image pair method for finding transition states," Journal of Chemical Theory and Computation **19**, 2410–2417 (2023).
